# Supplementary material for: Empanelment of the Population to the Primary Medical Care Institution of Sri Lanka: A Mixed-Methods Study on Outcomes and Challenges
Source: Healthcare (Basel). 2023 Feb 15;11(4):575. doi: 10.3390/healthcare11040575 (PMC9957292; doi:10.3390/healthcare11040575)
Supplement: Supplementary file 1 [file healthcare-11-00575-s001.zip › healthcare-2201898-supplementary.pdf]

Annex-S1: Proforma for extracting data on extent of registration and risk stratification

**Instructions for filling the form**:<br>1. Please enter name of the person completing the questionnaire in uppercase letters<br>2. Always type/write inside the boxes or lines provided.<br>3. In case of numerical responses, always enter only one digit in each box ☐<br>**Guide of the Interview:**<br>1. Good day! My name is ______________________.<br>2. We are here on behalf of [The International Union Against Tuberculosis and Lung Diseases and MOH Sri Lanka] conducting a survey of health facilities to assist the government in knowing more about health services in Sri Lanka.<br>3. Now I will read a statement explaining the study.<br>4. Your facility was selected to participate in this study. We will be asking you questions about empanelment of this PMCI under Primary Healthcare System Strengthening Project (PSSP) of Sri Lanka.<br>5. Neither your name nor that of any other health worker respondents participating in this study will be included in the dataset or in any report; however, there is a small chance that any of these respondents may be identified later. Still, we are asking for your help to ensure that the information we collect is accurate.<br>6. You may refuse to answer any question or choose to stop the interview at any time. However, we hope you will answer the questions, which will benefit the better understanding of extent of empanelment.<br>7. If there are questions for which someone else is the most appropriate person to provide the information, we would appreciate if you introduce us to that person to help us collect that information.<br>8. At this point, do you have any questions about the study? Do I have your agreement to proceed?		
A	General Information	
A1	Name of the person completing the questionnaire	______________________
A2	Date:	DD ☐☐ MM ☐☐ YYYY ☐☐☐☐
A3	Name of the respondent	______________________

A4	Designation of the respondent/s	________________
A5	Facility code:	☐☐☐☐☐
A6	Name of the facility	________________
A7	Location of the facility	________________
A8	Region/Province	________________
A9	Sub county/ district	________________
A10	Type of PMCI (Please tick ✓)	1. Divisional Hospital ☐ 2. PMCU ☐
A11	Area (Please tick ✓)	1. Urban ☐ 2. Rural ☐
A12	Service hours	1. 24x7 ☐<br>2. Forenoon only ☐<br>3. Forenoon and afternoon ☐
A12	Number of Grama Niladharies (GN) under this PMCI	☐☐
A13	Remarks (if any)	
B	Empanelment	
B1	Whether the catchment area is identified for this PMCI	1. Yes ☐ 0. No ☐
B2	Identified secondary or tertiary care institutions as referral centre	1. Yes within province only ☐<br>2. Yes within & also outside province ☐<br>0. No ☐
B3	Whether Personal Health Number has been issued to the people in the catchment area of PMCI	1. Yes, issued for whole population ☐<br>2. Yes, issued for part of the population ☐<br>0. No, not initiated issuing of PHN ☐
B3.i.	If the response for B4 is "0", what is the reason for not issuing PHN	________________

B4	Method of inviting people for registration<br><br>(multiple options possible)	1. Invitation letter to households ☐<br>2. Providing appointments ☐<br>3. Announcements ☐<br>4. Display of posters ☐<br>5. Others, Specify<br>____________________
B5	Type of registration process for enrolling people for PHN<br><br>(multiple options possible)	1. Active process – house to house ☐<br>2. Screening and enrollment at camp ☐<br>3. Passive (only when the individual asks) ☐<br>4. Opputunistic (when people visits hospitals/ clinics ☐<br>5. Mobile registration ☐<br>6. Staggered registration (for specific population groups) ☐<br>7. Volunteer/ paid temporary employee ☐<br>8. Night/ evening clinics ☐<br>9. Others<br>____________________
B6	Master list of households and people living in the catchment area is available at the PMCI	1. Yes ☐<br>0. No ☐
B7	Whether the above master list is updated last year	1. Yes ☐<br>0. No ☐
B8	Whether paper-based PHR available?	1. Yes ☐<br>0. No ☐
B9	Whether paper-based PHR issued to those registered?	1. Yes, to all ☐<br>2. Yes, partially ☐<br>0. No ☐

Annex-S2: Interview guide for understanding empanelment and its challenges

Respondent: Program Manager/Medical Doctor

Can you please elaborate on the empanelment process adopted by the PMCI	Probe the process of the empanelment, healthcare workers from PMCI, Volunteers, work plan.
What kind of challenges were identified while defining the catchment area	Probe for the defining the geographic location, overlapping borders, migrant population, slum areas
How the beneficiaries were contacted/ informed? What kind challenges were faced while identifying the catchment population and approaching them for empanelment	Probe for the process for population identification, awareness creation activities, provision of information and necessity of empanelment to the population, challenges faced by the health workers from the population, resistance if any, methods to reach working population
What were the challenges while establishing the network of laboratories	Probe for issues in identification of referral laboratory, private laboratories, patient identification number, barcoding of samples if any, measures to reduce the time delay to obtain the results
What were the challenges faced while issuing the PHN number	Probe for proper id card from patient, recognition or acceptance of patient /id by the software, network connectivity, location of empanelment centres, issues in generation of PHN number in the server
What were the challenges faced while issuing the PHR	Probe for availability of hard copy of PHR, adequate space to mention the details of the patient
What kind of challenges faced in management of routine work at PMCI during the empanelment process?	Probe for disruption of routine healthcare services due to less man power or delay in adaption of PSSP project at the PMCI

Respondent: Individual empanelled in the PMCI

Can you please tell us how did you come to know about the empanelment	Probe for the mechanism of contact with the empanelment team, support from the empanelment team, provision of necessary information before empanelment
Can you please elaborate on the empanelment process you have undergone at the PMCI	Probe for the process of the empanelment, ease of the process, and empanelment of family member, any challenges related to empanelment, how the procedure can be improved or made more patient friendly
What kind of challenges you have faced in the empanelment centre	Probe for the number of visits made to complete the empanelment, waiting time, place for waiting, access to the empanelment centre, network, staff cooperation, how to improve these challenges.
In your opinion, what will be the benefits of having PHN number	Probe for receiving of PHN number, understanding about the PHN number and advantages of having PHN number
How was your experience at the PMCI after receiving PHN compared to the period when PHN was not available	Probe for receiving PHR, challenges in registration for receiving healthcare services, challenges and experience during referral for laboratory services or apex hospital for further management, if any, compare to prior to PHN time
In your opinion what is the utility of PHR	Probe for utilization during emergency care, self-awareness regarding the treatment undergone, challenges related to receiving health care services, if any how easy it was to receive health care services when anyone forgot to bring their PHR to the PMCI
**Respondent: Individual not yet registered in the PMCI**	
Can you please tell whether you are aware of empanelment process going on at the PMCI under improvement of health services project	**Probe for the contact with the empanelment team, empanelment related information from other sources, support from the empanelment team and provision of necessary information empanelment**
What are the reasons for not undergoing empanelment	Probe for the initiation of activities for the empanelment, any other family members were empanelled, what are the challenges to plan for empanelment, any restrictions, or opinion against empanelment, staff cooperation, any challenges related to empanelment
